# School’s Out, Rules Out: Changes in parent rules and routines over summer from the What’s UP (Undermining Prevention) with Summer Observational Cohort Study

**DOI:** 10.21203/rs.3.rs-9175455/v1

**Published:** 2026-04-02

**Authors:** Emily Eglitis, Sarah Burkart, Christopher Pfledderer, Elizabeth L. Adams, R. Glenn Weaver, Bridget Armstrong, Keith Brazendale, Xuanxuan Zhu, Alexander McLain, Gabrielle Turner-McGrievy, Russell Pate, Andrew Kaczynski, Amanda Fairchild, Brian E. Saelens, Hannah Parker, Amy L. Yaroch, Carol Maher, Michael Beets

**Affiliations:** A Alliance for Research in Exercise, Nutrition and Activity (ARENA), Adelaide University, Adelaide, SA, Australia; B Arnold School of Public Health, University of South Carolina, Columbia, SC, USA; C UTHealth Houston, School of Public Health, Austin TX, USA; D Department of Health Sciences, University of Central Florida, Orlando, FL, USA; E College of Arts and Sciences, University of South Carolina, Columbia, SC, USA; F Seattle Children’s Research Institute; G Department of Health and Exercise Science, College of Health and Human Sciences, Colorado State University, Fort Collins, CO, USA; H Center for Nutrition & Health Impact, Omaha, NE, USA

## Abstract

**Background:**

Children engage in less healthful behaviors during the summer compared to the school year, when they are exposed to a highly structured school environment. It is unclear whether changes in children’s health behaviors may also be explained by parents relaxing rules/routines in summer (e.g., related to sleep, screens, diet). If so, this represents potentially modifiable drivers of children meeting health behavior guidelines. The purpose of this study was to determine if parents have different rules/routines in summer compared to the school year.

**Methods:**

This study used data from the What’s UP with Summer three-year, longitudinal observational cohort study (2021–2023) that followed elementary-aged children from 17 elementary schools in the southeastern United States. Parents of 1,084 children (age range 5–14 years, 48% girls) completed surveys during school (April/May) and summer (July) each year, yielding six total timepoints. Survey items assessed parental rules related to dietary behaviors (6 questions), screen-use (weekdays and weekends, 3 questions each), and sleep (weekdays and weekends, 3 questions each). Lower scores reflected fewer rules/routines that supported these health behaviors. Mixed-effects models examined changes in parenting rules from school year to summer controlling for socioeconomic status (poverty-income ratio, parent education), child age and sex.

**Results:**

Compared to the school year, parent rules in summer decreased for weekday screen-use (*b*= −0.31, 95% CI −0.34, −0.28), weekend screen-use (*b*= −0.09, 95% CI −0.13, −0.06), weekday sleep (*b*= −0.81, 95% CI −0.84, −0.78), and weekend sleep (*b* = −0.21, 95%CI −0.24, −0.17). There was no significant change in diet-related rules (*b*= −0.01, 95% CI −0.02, 0.00). Age had a significant interaction effect for screen and sleep rules/routines, while povertyincome ratio and child sex did not.

**Conclusions:**

Parents reported fewer rules for screen use and sleep during summer compared to the school year, and overall rules/routines declined as children grew older. The gap between summer and school sleep rules also widened with age. These findings suggest that the structure of school year routines help support parents in maintaining rules and routines that shape children’s health behaviors.

## Introduction

Children experience accelerated weight gain during the summer vacation/holidays compared to the school year^1^. This appears to be driven by different time-use behaviors between holidays and school days. Growing evidence indicates that away from school on holidays and weekends, children often display poorer diets, less moderate-to-vigorous physical activity, increased sedentary and screen behaviors, and altered sleep patterns^1–6^. Patterns for dietary and sleep behaviors is less clear^1^, although evidence suggests that during school holidays children consume more unhealthy foods and drinks and less fruit and vegetables^7^. Over holidays, children may get more sleep^8^, but may achieve this with later sleep/wake patterns compared to the school year^9–11^. Unhealthy behavior changes are evident even in contexts with relatively short two-week school holidays^4^. Since the summer holidays are the longest, consecutive period where children are away from school, unhealthy behaviors can potentially have the biggest cumulative effects that result in accelerated weight gain.

The school day is inherently structured, with pre-planned, segmented and adult-supervised components that promote physical activity (e.g., through physical education lessons, sport, or play at recess), limit opportunities for recreational screen use and snacking^2^, and help maintain consistent sleep schedules with regular school start times. Unstructured non-school days allow children to have greater autonomy over their choices, and when given more freedom, children tend to display healthy behaviors, such as consuming less healthy snacks^3^, which may be the result of child preferences or the types of food available in the home. Additionally, outside of the school environment, family rules and routines help to create order and streamline daily life^12^. Substantial evidence indicates family rules/routines are associated with better child health behaviors^12–14^. For example, health-promoting rules/routines can support children in establishing healthy behaviors that reduce the risk of overweight and obesity, such as screen-use rules that are associated with increased physical activity^15^ and diet-related rules that support healthier eating behaviors^15,16^. Bedtime rules that promote regular bedtimes and the removal of electronic devices from bedrooms can improve children’s sleep duration and quality^17^. However, existing data on family rules and routines are typically collected during the school year, assuming consistency year-round.

These less-healthy time-use patterns in the summer holidays may reflect reduced daily structure in the absence of school, however qualitative evidence^18^ suggests parents may also relax rules/routines during the summer holidays when the routines and pressures of the school day are absent. To our knowledge, very limited evidence exists on how health-related parent rule/routines change over time as children get older, or if rules/routines are different for boys versus girls. Understanding if changes in context (i.e., school term to summer holidays) influence parenting rules/routines may improve our understanding of key drivers behind factors related to child overweight/obesity and identify new intervention opportunities.

Therefore, the aim of this study was to examine differences in parents’ health-related rules/routines between the school year and summer in a diverse cohort of children over three years. Specifically, the study questions were:

1. Do health-related rules/routines differ in the summer holidays compared to the school year?
2. If so, do the differences in rules/routines between school and summer vary by child age, child gender, or socioeconomic status?

## Methods

### Participants

This study used data from the What’s UP (Undermining Prevention) with Summer study (National Institutes of Health R01DK116665, WUP) designed to understand summer effects on unhealthy weight gain among elementary-aged children in the United States. The reporting of this study conforms to the STROBE checklist^19^ (Supplementary file 1). This study was approved by the author’s (MB) Institutional Review Board (Pro00080382) and is described elsewhere^5^. Briefly, the design was a longitudinal observational cohort that followed elementary-aged children (5–12 years) across three years (2021, 2022, and 2023). Children were recruited from 17 elementary schools in a mid-sized metropolitan area in South Carolina in the southeastern United States. Recruitment occurred via the child’s school, with parents providing informed consent and children providing assent prior to participation.

To account for parents/children electing to discontinue in the study, we refreshed the sample^20–22^ in the early spring (February/March), prior to school data collection in April/May each year. Refreshing the sample involved replacing students who dropped out of the study with students who had similar demographic characteristics. Refreshing is methodologically consistent with other large-scale cohort studies^21–23^. Criteria of the refreshment sample was to ensure similar proportions of students were present in the study based on child socio-demographics (i.e., biological sex, race/ethnicity, household income) from the initial year of data collection in 2021 and to replace children based upon grade as the cohort aged over time.

### Child and household characteristics

At the school measurement timepoint each year, parents completed an online survey via their smartphone about the demographics of their child (biological sex and age), information about total annual household income, and the number of people (adults and children) living in their home. This information was used to calculate the ratio of poverty to income for each year according to U.S. Federal Poverty Guidelines established by the Department of Health and Human Services^24^.

### Health behaviors

Parents received a text with a link to a HIPAA-compliant web-based survey platform and completed the survey on their smartphone twice each year – during school (April/May) and again during summer (mid-July) for a total of 6 timepoints. Surveys included health-related questions from the Child Routines Inventory Daily Living Routines Subscale^25^ and items assessed parental rules/routines^26–30^. Parents received a $25 gift card for completing the survey. The average completion time was 25 min. The sleep, screentime, and meal rules/routines were adapted from existing surveys and the questions asked can be found with the results in Table 2. Where parents had more than one child enrolled in the study, they completed separate surveys for each child.

Sleep items included 3 questions regarding bedtime, consistency of bed timing, and when a child must wake^17,31–34^. The screentime items included 3 questions regarding rules/routines allowing the child to watch/use screens, setting limits on how much time a child can use screens, and how often the parent tells the child to turn off screens^35^. These questions were phrased to capture rules/routines parents have during weekdays (Sunday through Thursday) and weekend nights (Friday and Saturday) nights on a 4-point Likert scale ranging from 0 “never” to 3 “always”. The survey also asked 6 questions about rules/routines around meals. These questions were asked about a typical week that includes both weekdays and weekend days (see Table 2 for the specific items). Individual items were coded such that higher scores indicate greater extent of rules/routines. For the purpose of this study, the individual items for each behavior were averaged to create a composite “rules/routines” for sleep, screentime, and meals, separately, with alphas ranging from 0.63 to 0.87 across composites. Scores for summer holidays versus the school year were calculated separately. Furthermore, composite “rules/routines” scores were created for sleep and screentime on weekdays and weekend days, separately. Mean scores were calculated for each construct (e.g., mean of all diet rules, mean weekday screen rules, mean weekend screen rules, etc.). Positive values represent more rules/routines in the summer, and negative values represent a reduction in the average rules/routines parents have during summer compared to school.

### Sample size

The target sample size of 1083 was based upon the primary aim of the WUP study. A post hoc power analysis was conducted based on the research question guiding this secondary analysis. It suggested that a sample size of n=320 provided 80% power to detect a difference of d=0.42 in the rule/routines outcomes. Accounting for an expected 20% attrition per year across the 3 years, this suggested an initial enrollment sample size of 504 provided excellent power for the current analysis. The original study was planned to start spring 2020. Due to COVID-19 restrictions, the study began in 2021.

### Statistical analyses

Linear and non-linear mixed-effects models with random intercepts and slopes, clustering for repeated measures on children, nested within families, within schools, were used to examine changes in parenting rules/routines between the school year and summer. Non-linear models included non-linear age terms. Socioeconomic status (income–poverty ratio, parent education), child age, and sex were entered as fixed effects. Moderating effects were examined by including interaction terms between summer (vs. school) and child age, sex, and poverty-income ratio. This approach tested whether changes in parental rules/routines differed across demographic groups. Analyses used full information maximum likelihood estimators, with cases excluded listwise when all response to the rules/routines questions were missing. Loss to follow-up was managed by refreshing the cohort annually to offset attrition. Sensitivity analyses compared results from linear and non-linear models across outcomes to assess robustness. All analyses were performed using Stata (v.18, College Station, TX). Coefficients were converted to standardized mean differences using the pre-test and post-test values and the pooled standard deviation (Cohen’s *d*) to facilitate clinical interpretation, using accepted benchmarks of <0.2 = trivial, 0.2–0.5 = small, 0.5–0.8 = medium, and ≥0.8 = large^36^.

## Results

### Participants

Parents of 1,084 children contributed data across three summers and school years. Children had a mean age of 9.8 years (SD 1.8), and 49% were female (Table 1). Children were present in the cohort for an average of 3.7 (range 1 to 6) assessment periods (total 3 school and 3 summer assessment periods).

### Main results

**Figure 1.**
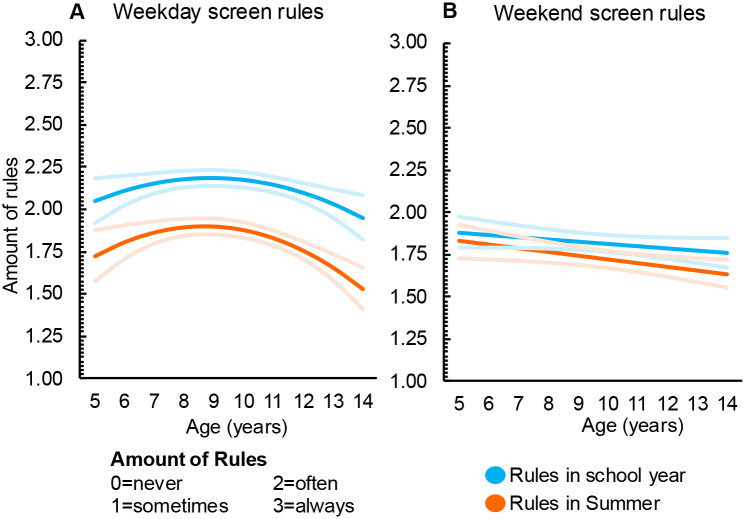
Changes in parent screen rules/routines across weekends, weekdays, summer holidays and the school year (n=1086) Figure 1: Parent rules and routines for screen-use on weekdays (a) and weekend days (b). Red values indicate the school year, blue indicates the summer holidays. Light shading illustrates the 95% confidence intervals.

**Figure 2.**
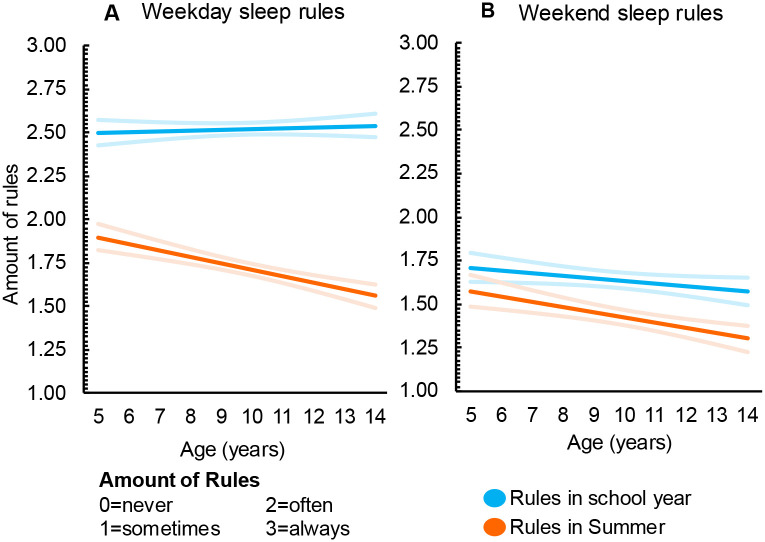
Changes in parent sleep rules/routines across weekends, weekdays, summer holidays and the school year (n=1086) Figure 2: Parent rules and routines for sleep on weekdays (a) and weekend days (b). Red values indicate the school year, blue indicates the summer holidays. Light shading illustrates the 95% confidence intervals.

Results for the main analysis are presented in Table 2 and Figures 1 and 2. Compared to the school year, parent rules/routines in summer decreased for weekday screen-use (*b*= −0.31, 95% CI −0.34, −0.28) (Figure 1a), weekend screen-use (*b*= −0.09, 95% CI −0.13, −0.06) (Figure 1b), weekday sleep (*b*= −0.81, 95% CI −0.84, −0.78) (Figure 2a), and weekend sleep (*b*= −0.21, 95%CI −0.24, −0.17) (Figure 2b). There was no significant change in diet-related rules/routines between summer and school (*b*= −0.01, 95% CI −0.02, 0.00) (not depicted).

### Moderator analyses

Separate moderator analyses were undertaken to determine if outcomes varied according to child age, sex, or income (poverty to income ratio). Age had a significant interaction effect for screen and sleep rules/routines (Figures 1 and 2). Diet rules/routines did not change as children got older (***b***= −0.01, 95% CI −0.01, −0.00) (not depicted). No other sociodemographic characteristics were statistically significant moderators.

Age had a non-linear effect on screen rules/routines. On weekdays, rules/routines in both summer and school periods initially increase and then decrease in early adolescence (Figure 1a). On weekends, screen rules/routines were similar between school and summer and decreased with age (Figure 1b).

Age also had a non-linear effect on sleep rules/routines Weekday sleep rules/routines in the school year remained consistent across age groups, but rules/routines decreased with age in the summer (Figure 1c). Weekend sleep rules/routines also decreased with age, but more dramatically in the summer (Figure 1d). This means that for all sleep rules/routines, there was a widening summer-school rule gap as children age.

While girls had slightly more diet rules/routines over summer than boys (*b=* 0.03, 95% CI 0.00, 0.05), and middle-income families had fewer weekday sleep rules/routines than low-income families (*b=* −0.11, 95% CI −0.19, −0.04), overall, there were no consistent patterns regarding differential effects by child sex or family income.

## Discussion

This study investigated whether parents applied different levels of health-related rules and routines during summer compared with the school year, and whether patterns emerged according to child age, gender, or socioeconomic status. No previous studies have examined the potential role of parent rules/routines in understanding the differential in health behaviors that many studies have observed between the school year and summer holidays. The key findings of this study were that parents implemented less household rules and routines related to screen use and sleep during summer compared with the school year. These summer–school differences were most pronounced on weekdays, and the gap in sleep-related rules/routines widened as children grew older. In contrast, no significant changes were observed in diet-related rule behaviors, and there were no differential effects by gender or socioeconomic status. The quality and consistency of rules and routines support the health and wellbeing of all family members and influence how family members interact with their environment^6^.

Rules and routines can provide predictability and stability to the family environment, which can help to reduce conflict and foster harmony^37,38^. Consistent routines have been linked to lower stress and risk behaviors in children, fewer depressive symptoms in parents and their children, improved marital satisfaction, and better social-emotional functioning in young children^37,39,40^. There could be long-lasting consequences for youth too, with evidence that adolescents exposed to low family rules and routines have poorer diet and exercise behaviors ten years later^41^.

Findings from this study indicate that the existence of rules and routines around sleep and screentime behaviors shifts between school and summer in ways that parallel the behavioral changes consistently observed in children during summer, including reduced physical activity, increased sedentary and screentime, and less healthy dietary behaviors^5,8,42–46^. The lack of change for dietary rules/routines identified in this study is interesting, as evidence exists that children’s dietary behaviors are less healthy over the summer holidays compared to the school year^47,48^. This may be explained in part by imperfect nature of attempting to measure diet-related rules/routines. For instance, the items we measured may reflect family values that remain consistent throughout the year (i.e., the importance of eating a homecooked meal together, rather than by oneself). Perhaps other questions, such as weekly spending on discretionary food items, would better reflect parents’ attempts to manage children’s dietary behaviors. Diet-related rules/routines may in fact not change, but simply have greater exposure to the home-environment which may be less restrictive than the school day. Overall, these findings provide novel evidence that parent rules/routines fluctuate between the school term and school holidays, in response to children’s exposure to structure, rather than being static. In particular, they support the conceptual model of the Structured Days Hypothesis, which posits that segmented, pre-planned, and supervised activities external to the home help to anchor healthy routines within the home^2,49^. When children attend structured settings such as school, parents appear better able to maintain consistent health-related rules and routines within the home^45^. In contrast, during summer, when such external structure is largely absent and children spend more time at home, parents face greater responsibility to regulate children’s behaviors.

Changes in the level of parental rules and routines during summer may reflect both parental values and the external pressures families face. For some families, a relaxation of rules and routines may be seen as a reward or release from the rigid demands of the school year, including the early wakeups, tightly scheduled routines, schoolwork, and extracurricular activities^18^. Parents may view the holidays as a time for children to enjoy greater autonomy and flexibility, with less emphasis on strict regulation. At the same time, maintaining rules requires consistent effort and can generate conflict, and parents themselves may experience fatigue from this responsibility^50^. The lack of summertime rules and routines may also represent a means of strengthening relationships. The summer period may therefore represent not only a break for children but also a reprieve for parents, in which relaxing rules reduce daily strain and allow for a more harmonious household environment.

Alternatively, changes in parental rules and routines may reflect the multiple demands on parents’ time and attention, often compounded by limited financial or social support^51^. When children are home during summer, the responsibility for structuring daily routines and managing time-use falls more heavily on parents, increasing both logistical and emotional load. In this context, a reduction in rules and routines may signal decreased capacity rather than diminished concern for children’s wellbeing or parents purposefully decreasing their rule behavior. In this case, these findings could underscore the need to provide parents with support to maintain health-promoting routines. Consistent with this perspective, the U.S. Surgeon General has highlighted that parents cannot be expected to shoulder the full responsibility for children’s health behaviors in isolation, and that broader societal supports are needed to enable effective parenting practices^51^. However, supporting parents and families to maintain rules and routines is challenging. Parents are difficult to recruit and retain in interventions, and participation in such interventions can create additional burdens, especially for mothers^52^. Approaches must therefore be sensitive to family capacity, emphasizing simple, sustainable routines. Indirect support, such as reminder tools, community-based activities, or peer networks, may reduce the burden on parents. Interventions should also consider broader household impacts, including workforce participation and redistribution of responsibilities^52^.

The presence of external structure appears to support parents in enforcing healthier rules and routines around sleep and screen use, suggesting that similar benefits of the school year may be achieved through alternatives such as structured programming in the summer. Childfocused interventions and programs in the summer play a critical role in this regard, not only by directly engaging children but also by easing the burden on parents through practical tools, structured activities, and guidance that reinforce consistent rule-setting in the home. Unfortunately, summer activities can be prohibitively expensive for families and access to structured summer opportunities is often inequitable^53,54^. Families who may benefit most from these supports are frequently the least able to access them, particularly due to the high costs associated with formal programs^55^. This gap underscores the need for policy approaches that ensure affordable, accessible options for all families, particularly those with limited resources.

A strength of this study is that it was also conducted in a large, diverse cohort of children over three years, which strengthens the robustness and generalizability of the findings. There are also several limitations to consider. Potential sources of bias included selection bias from attrition and social-desirability bias due to parent self-report. Parents may have over-reported the extent to which health-related rules and routines exist, meaning that absolute levels of rules/routines may be inflated, although relative differences between summer and school periods are likely still preserved. Measuring and interpreting complex constructs such as rules and routines is inherently challenging^56^. The use of four-point Likert scale items, while appropriate for capturing broad patterns, makes clinical interpretation less precise. For the relatively abstract construct of “amount-of-rules”, it is challenging to determine the clinical relevance of the effect sizes observed. Although small in size, the consistency in the direction of effects across multiple behavioral domains indicates that the patterns reflect a genuine phenomenon rather than measurement error or chance variation.

These findings suggest that there is a beneficial role of rules/routines in the family home that extends beyond behavior regulation and supports children’s health and wellbeing. This is supported by a recent systematic review with narrative synthesis, that found children’s routines are positively associated with a wide range of outcomes spanning cognitive development, socio-emotional wellbeing, academic achievement, mental health, and physical health^12^. However, the impact of summer holiday versus school year changes in household rules and routines on children’s actual behaviors remains unclear. This study also found changes in rules/routines as children aged. It is considered common and developmentally appropriate for rules and routines to evolve as children age^17,56^. However, it is not clear how rules/routines can best evolve appropriately in a manner that supports, rather than undermines, healthy behaviors. Exploring these questions could provide valuable insights to guide parent education and support strategies.

This study provides several avenues for future research. Future studies should examine the association between parental rule behaviors and objective measures of children’s physical activity, sedentary time, and screen use to determine whether observed seasonal changes in rules translate into meaningful shifts in health and wellbeing. It would be useful to explore if other forms of external structure during summer, for example summer programs, have the same effect in modifying parental rules and routines.

Finally, these findings prompt us to consider how child-focused interventions impact parents and families more broadly. Positioning parenting rules/routines as one potential pathway through which external structure shapes children’s health behaviors adds nuance to current models of understanding summertime weight gain. Interventions that have aimed to mitigate accelerated weight gain^57^ may inadvertently help families continue with consistent, health-supporting rules/routines when school is not in session. Understanding the ripple effects that child-focused interventions have on families has implications for the design of effective, sustainable interventions that address both child behavior and parental capacity to support health-promoting routines.

## Conclusion

Consistent with the Structured Days Hypothesis, parents display fewer rules and routines around children’s sleep and screentime use during summer compared with the school year, with differences most evident for weekdays. The level of rules/routines declined as children aged, and the gap between school and summer sleep rules and routines widened with age, suggesting that older children and adolescents may be particularly susceptible to seasonal disruption in health behaviors. In contrast, no systematic changes were observed in the diet-related rules/routines that we measured, and findings did not vary by sex or socioeconomic status. These findings indicate that family rules/routines are potential modifiable factors through which structured settings outside the home, such as school, shape children’s daily health behaviors. When such structures are absent during summer, parents face greater responsibility to manage children’s health behaviors but may lack the capacity or resources to maintain supportive rules/routines. These challenges indicate that holiday interventions, such as equitably accessible summer programs, show promise in supporting parents in sustaining healthy rules and routines throughout the year. Such an approach could play a meaningful role in mitigating seasonal weight gain.

## Supplementary Material

Supplementary Files

This is a list of supplementary files associated with this preprint. Click to download.

- Eglitis2025ParentrulesSTROBEchecklistcohort.docx

## Figures and Tables

**Table 1.** Participant demographics

	Overall (n=1086)		2021 (n=702)		2022 (n=718)		2023 (n=738)	
**Child**								
Females	49%		50%		50%		48%	
Age (years, SD)	9.8	±1.8	8.9	±1.7	9.6	±1.8	10.8	±1.6
PIR (percentage, SD)	219	±104.9	229	±110.6	219	±107.8	211	±95.7
Race/Ethnicity								
Hispanic	8%		6%		9%		8%	
Black	34%		32%		35%		31%	
White	47%		53%		45%		50%	
Multi	8%		7%		8%		9%	
Other	3%		2%		3%		2%	
**Parent**								
Education								
Less than college	32%		29%		30%		28%	
2–4yr college	40%		44%		40%		40%	
Graduate	28%		28%		30%		32%	

Note. Graduate: Masters/PhD qualification, PIR: Poverty-income-ratio, other: Asian, Native American

**Table 2.** Parent rules and routines in summer compared to the school year (n=1086)

Rule	Coefficient (*b*)	SE	95% CI		SDM (d)	SE
**Diet Composite**	**−0.01**	0.01	−0.02	0.00	0.04	0.03
Eat at a restaurant or fast food for dinner (such as McDonald’s, Bojangles’, Dom)	0.00	0.01	−0.03	0.02	0.03	0.03
Eat dinner while watching TV/screen	**−0.03**	0.01	−0.06	0.00	0.08	0.03
Let your child eat sweets (such as candy, ice cream) for snack	**−0.03**	0.01	−0.06	0.00	0.07	0.03
Allow your child to take food or beverages into their bedroom	0.05	0.01	0.03	0.08	0.12	0.03
Family eats dinner together	0.02	0.02	−0.01	0.05	0.04	0.03
Eat dinner that is prepared at home	**−0.06**	0.01	−0.09	−0.03	0.13	0.03
**Screentime Composite - Weekday**	**−0.31**	0.02	−0.34	−0.28	0.56	0.03
Limits or have rules on any screens/electronic devices on weekday	**−0.39**	0.02	−0.43	−0.35	0.54	0.03
Rules for turning off screens/electronic devices on weekday	**−0.18**	0.02	−0.22	−0.13	0.24	0.03
Allow to watch any screen/use an electronic device on weekday	**−0.36**	0.02	−0.40	−0.32	0.50	0.03
Rule for how late your child can watch screen/use an electronic device on weekday	**−0.27**	0.03	−0.32	−0.22	0.30	0.03
**Screentime Composite - Weekend**	**−0.09**	0.02	−0.13	−0.06	0.14	0.03
Limits or have rules on any screens/electronic devices on weekend	**−0.11**	0.02	−0.16	−0.07	0.15	0.03
Turn off screens/electronic devices on weekend	**−0.09**	0.02	−0.13	−0.04	0.12	0.03
Allow to watch any screen/use an electronic device on weekend	**−0.10**	0.02	−0.14	−0.05	0.13	0.03
How late is allowed to watch screen/use an electronic device on weekend	−0.04	0.03	−0.10	0.01	0.04	0.03
**Sleep Composite - Weekday**	**−0.81**	0.02	−0.84	−0.77	1.23	0.04
Set limits or rules on when your child(ren) must go to bed on weekday	**−0.68**	0.02	−0.72	−0.64	1.03	0.04
Child(ren) go to bed at the same time at night on weekday	**−0.63**	0.02	−0.66	−0.59	0.96	0.04
Set limits or rules on when your child(ren) must wake up on weekday	**−1.11**	0.03	−1.16	−1.06	1.35	0.04
**Sleep Composite - Weekend**	**−0.21**	0.02	−0.24	−0.17	0.32	0.03
Set limits or rules on when your child(ren) must go to bed on weekend	**−0.19**	0.02	−0.23	−0.14	0.25	0.03
Child(ren) go to bed at the same time at night on weekend	**−0.11**	0.02	−0.15	−0.07	0.16	0.03
Set limits or rules on when your child(ren) must wake up on weekend	**−0.32**	0.03	−0.37	−0.27	0.39	0.03

Note. Items are coded so that higher values reflect more/healthier rules/routines in summer compared to the school year. Positive values indicate an increase in rules and negative values indicate a decrease in rules and therefore less healthy behaviors (red text). Values in bold are statistically significant (P≤0.05).

## Data Availability

The datasets generated and/or analyzed in the current study are not publicly available because additional analyses are ongoing. Data will be made available from the corresponding author on reasonable request at the time the primary outcome papers are published.
